# Mid-term outcomes of endoscopic vein harvesting in coronary artery bypass grafting: a retrospective cohort study

**DOI:** 10.1186/s13019-024-02930-5

**Published:** 2024-06-27

**Authors:** Wuwei Wang, Yiming Liu, Haoyu Qi, Yafeng Liu, Yunfei Jiang, Rui Fan, Junjie Shao, Wen Chen, Cunhua Su, Xin Chen

**Affiliations:** Department of Thoracic and Cardiovascular Surgery, Nanjing First Hospital, Changle Road 68, Nanjing, China

**Keywords:** Endoscopic vein harvesting, Open vein harvesting, Coronary artery bypass grafting, Prognosis

## Abstract

**Objectives:**

Endoscopic vein harvesting (EVH) is an alternative technique to obtain the saphenous vein for coronary artery bypass grafting (CABG) surgery. We aimed to evaluate the early and mid-term outcomes of patients with EVH in CABG.

**Methods:**

This cohort study included consecutive isolated CABG patients in Nanjing First Hospital from July 2020 to December 2022 using propensity score matching methods. Patients were classified to EVH group and open vein harvesting (OVH) group according to the vein harvesting methods. The primary outcome was the all-cause death, and the secondary outcomes were major adverse cardiovascular events (MACEs) including cardiovascular death, heart failure, myocardial infarction and revascularization and asymptomatic survival in the follow-up.

**Results:**

Totally 1247 patients were included in the study with 849 in OVH group and 398 in EVH group. Patients with EVH were more female, diabetes, higher body mass index, more multi-vessel and left main diseases. 308 pairs were formed after the matching. There was no significant difference in the rates of in-hospital death (EVH vs. OVH, 2.3% vs. 1.3%, *P* = 0.543). During the 3 years follow-up, EVH grafts were considered not inferior to OVH grafts, no differences were found in all-cause death [8.5% vs. 5.0%, hazard ratio (HR) 1.565, 95% confidence interval (CI): 0.77–3.17, *P* = 0.21], MACEs (8.1% vs. 7.1%, HR 1.165, 95CI: 0.51–2.69, *P* = 0.71) and asymptomatic survival (66.7% vs. 72.5%, HR 1.117, 95%CI: 0.65–1.92, *P* = 0.68).

**Conclusions:**

EVH grafts were considered comparable to OVH grafts in patients following CABG in the 3 years follow-up.

**Supplementary Information:**

The online version contains supplementary material available at 10.1186/s13019-024-02930-5.

## Backgrounds

Coronary artery bypass graft (CABG) surgery is a common procedure in the treatment of multi-vessel coronary artery disease [1]. From the data of Chinese Cardiac Surgery Registry, there are over 10,000 CABGs performed per year [2]. Although totally arterial CABG shown better prognosis in the long-term follow-up, the saphenous vein is still the most commonly used graft second only to the left internal mammary artery (LIMA) currently.

Open vein harvesting (OVH) was the traditional technique to obtain the saphenous veins for CABG surgery. Its simple operation and short learning curve are favored by surgeons, however meanwhile, OVH has several drawbacks including postoperative pain, wound complication and increased risk of infection [3–5]. Endoscopic vein harvesting (EVH) has become an alternative technique and widely used in decades due to its substantial decrease in postoperative leg wound complications. The ESC/EACTS [6] and ACC/AHA/SCIA [1] guidelines for coronary artery revascularization both recommend the use of EVH in CABG to reduce the wound complications.

In the previous studies, the EVH technique has been shown to significantly reduce wound-related complications, decrease postoperative pain and increase patient satisfaction comparing with OVH [5]. A large randomized controlled trial (REGROUP Trial) on western countries showed no significant differences in the risk of major adverse cardiac events [7]. However, its safety evidence in different race were still limited especially in Asian populations. Several researches had shown that endoscopic technique may cause the excessive stretching and potential tears or avulsions of saphenous vein, which incite early graft atherosclerosis and failure [8]. Therefore, the aim of this cohort study is to compare the outcomes of OVH and EVH in CABG surgery in the early and mid-term follow-up in Chinese population.

## Methods

### Patients

This retrospective cohort study included consecutive patients who underwent primary isolated CABG at Nanjing First Hospital, a level A tertiary hospital located in Jiangsu Province, China, between July 2020 to December 2022. The study was approved by the ethics committee of Nanjing First Hospital for the data collection (KY20170811-03) and the written informed consent was not required because of the nature of the study.

The exclusion criteria included the following: (1) concomitant procedures, including valve surgery, aorta surgery, congenital heart diseases surgery, Maze procedure, ventricular aneurysm surgery and other surgeries. (2) previous CABG procedure. (3) no saphenous vein use in the surgery. (4) missing vein graft data patients. All eligible patients were classified into EVH group and OVH group according to the different method of vein harvesting. Clinical data including medical history, procedural details and clinical outcomes were collected through the specific multi-center databases of Cardiovascular Surgery Department in Jiangsu Province.

### Surgical procedures

After general anesthesia, all patients received a median sternotomy. Skeletonized or pedicled LIMA was obtained as a graft to left anterior descending artery. The decision to perform either EVH or OVH procedure was taken individually by each treatment group. Patients with open vein harvesting were often made an incision started at the ankle. The subcutaneous tissue was dissected to expose the greater saphenous vein and then extended upward the skin incision along the course of the vein. While, a 2–3 cm transverse incision was made over the vessel at the knee for patients with endoscopic vein harvesting. Using a blunt conical as a dissecting tool to push tissues off the vein and then change it into a clamp to cauterize and divide the side branches. incisions were made separately at the groin and the ankle to divide the end of the vein. All EVH procedures were conducted by a specific surgeon with more than 2 years surgical experience and over 50 surgeries per year. The coronary artery anastomosis was conducted with or without cardiopulmonary bypass. Transit time flow measurement (TTFM) was used to control the quality of CABG before chest closure with mean arterial pressure ≥ 80 mmHg [9].

### Outcomes

The primary outcome of this study was all-cause mortality from the surgical time to the endpoint follow-up. The secondary outcomes were major adverse cardiovascular events (MACEs) and asymptomatic survival in the mid-term follow-up. MACEs were defined as the cardiac-cause death, heart failure, myocardial infarction and revascularization. The post-operative follow-up was conducted by Cardiovascular Surgery Follow-up Group in Nanjing First Hospital at 1,3,6,12 month and per year after the surgery. Direct telephone monitoring was used to confirm survival and MACEs for the final follow-up form April to June 2023. For those who lost to follow-up, the last recorded data were used.

### Statistical analysis

Continuous variables are presented as mean(SD) or median(interquartile range[IQR]) according to the data distribution. The student t test or Mann-Whitney U-test were applied to normally and non-normally distributed variables separately. Categorical data are presented as frequencies and percentages and were compared using chi-square test or Fisher’s exact probability method.

Propensity score matching(PSM) was used to reduce the impact of potential confounders. The propensity scores were calculated using logistic regression, taking into account the demographic and clinical characteristics mentioned in Table 1. Patients underwent OVH were matched to patients with EVH using the propensity score with 1:1 greedy nearest neighbor matching with a caliper of 0.02 [10]. Standardized mean differences(SMDs) < 0.1 were considered to be an indicator of ideal balance between groups [11]. This method functionally relied on the R package MatchIt.

**Table 1:** Baseline characteristics before and after PS matching between OVH and EVH group

	Before PS matching			After PS matching		
	OVH (*n* = 849)	EVH (*n* = 398)	SMD	OVH (*n* = 308)	EVH (*n* = 308)	SMD
Age, mean(SD)	64.55(9.58)	65.08(10.03)	0.053	64.13(9.57)	64.39(10.13)	0.026
Male, n(%)	630(74.2)	267(67.1)	0.152	215(69.8)	222(72.1)	0.048
BMI, mean(SD)	24.58(3.17)	25.14(3.39)	0.166	25.01(3.21)	25.05(3.33)	0.012
Smoke, n(%)	387(45.6)	188(47.2)	0.033	145(47.1)	148(48.1)	0.020
Hypertension, n(%)	584(68.8)	295(74.1)	0.122	220(71.4)	224(72.7)	0.030
Diabetes, n(%)	371(43.7)	188(47.2)	0.071	136(44.2)	144(46.8)	0.052
Hyperlipidemia	156(18.4)	144(36.2)	0.371	86(27.9)	73(23.7)	0.088
COPD, n(%)	29(3.4)	8(2.0)	0.100	9(2.9)	7(2.3)	0.046
PAD, n(%)	30(3.5)	30(7.5)	0.152	13(4.2)	16(5.2)	0.037
CAS, n(%)	88(10.4)	49(12.3)	0.059	29(9.4)	34(11.0)	0.069
Previous Stoke, n(%)	92(10.8)	53(13.3)	0.073	43(14.0)	40(13.0)	0.029
Previous PCI, n(%)	108(12.7)	38(9.5)	0.108	30(9.7)	32(10.4)	0.022
eGFR, mean(SD), ml/(min•1.73m^2^)	92.57(28.49)	91.75 (30.22)	0.027	93.77(30.18)	92.94(30.28)	0.028
eGFR < 90 ml/(min•1.73m^2^)	382(45.0)	184(46.2)	0.025	136(44.2)	139(45.1)	0.020
Angina, n(%)	738(86.9)	340(85.4)	0.486	263(85.4)	261(84.7)	0.018
ACS, n(%)	242(28.6)	206(52.6)	0.480	135(43.8)	137(44.5)	0.013
NYHA III-IV, n(%)	276(32.5)	86(21.6)	0.265	77(25.0)	77(25.0)	0.000
LVEF, mean(SD), %	58.01(9.96)	58.12(10.03)	0.011	58.45(9.32)	57.86(10.06)	0.059
LVEF < 50%, n(%)	188(22.1)	87(21.9)	0.007	66(21.4)	70(22.7)	0.031
3 vessel disease, n(%)	723(85.2)	380(95.5)	0.497	288(93.5)	290(94.2)	0.031
LM disease, n(%)	257(30.3)	144(36.2)	0.123	101(32.8)	105(34.1)	0.027
Emergency, n(%)	33(3.9)	16(4.0)	0.007	15(4.9)	15(4.9)	0.000
EuroSCORE II, mean(SD), %	2.00(2.39)	2.60(3.47)	0.174	2.04(1.93)	2.26(2.24)	0.065

Values are presented as mean(SD) or frequency(percentage)

PS: propensity score. OVH: open vein harvesting. EVH: endoscopic vein harvesting. SMD: standardized mean difference. BMI: body mass index. COPD: chronic obstructive pulmonary disease. PAD: peripheral arterial disease. CAS: carotid artery stenosis. PCI: percutaneous coronary intervention. eGFR: estimated glomerular filtration rate. ACS: acute coronary syndrome. NYHA: New York heart association. LVEF: left ventricular ejection fraction. LM: left main artery

Time-to-events were computed using Kaplan-Meier survival curves. Log-rank statistics was used to test the differences of mortality after the PSM cohorts. Because of the competing risk of mortality to observe the MACEs and asymptomatic survival probabilities, the Fine-Gray contrast method was used to compared the differences. Hazard ratios (HRs) and 95% confidences intervals (CIs) were used to assess the proportional hazard assumption.

All statistical analyses were performed using R software, version 4.2.2(http://www.r-project.org/). A two-side *P* value < 0.05 was considered statistically significant.

## Results

### Baseline characteristics

**Fig. 1:**
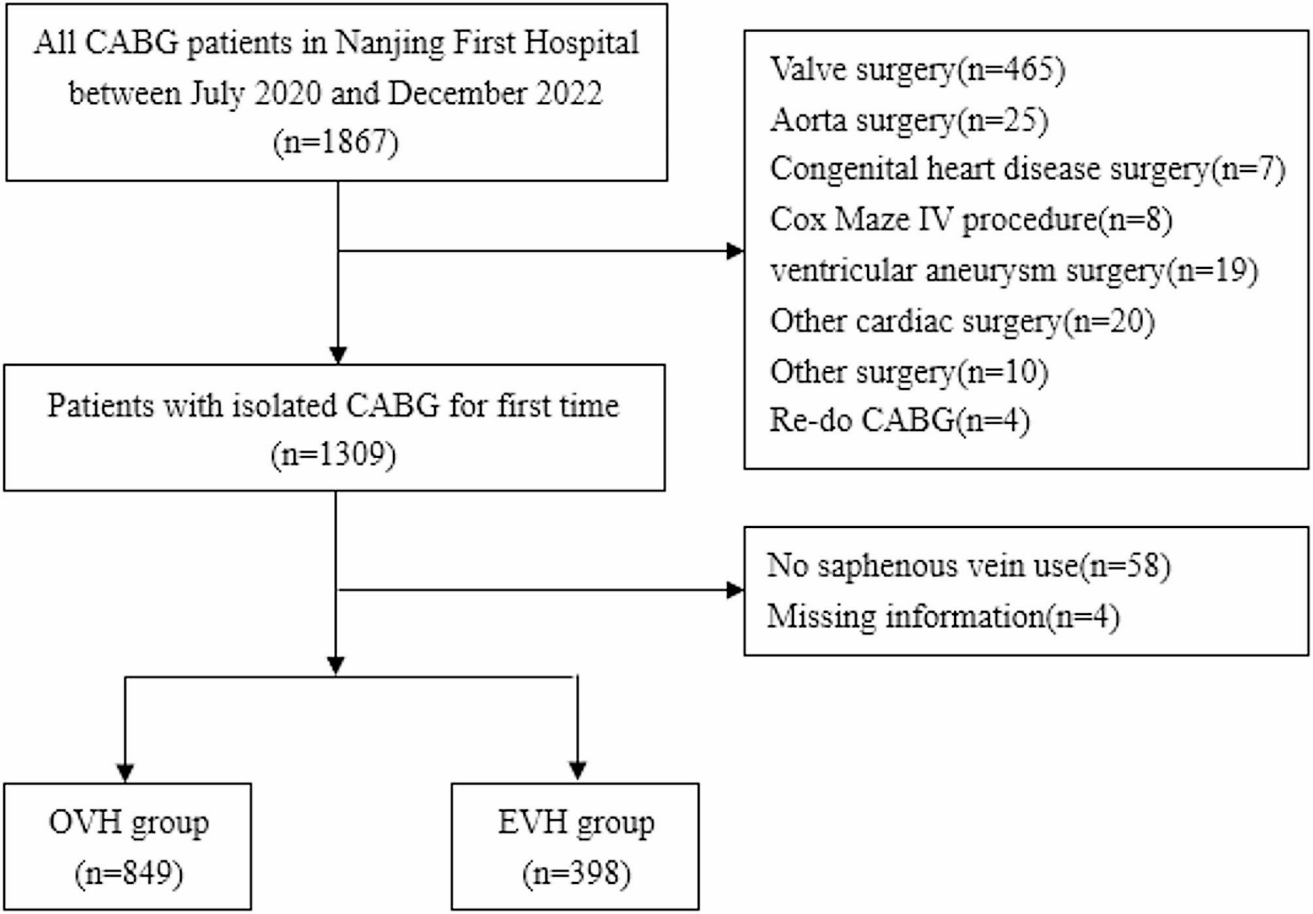
Flowchart of the study CABG: coronary artery bypass graft. OVH: open vein harvesting. EVH: endoscope vein harvesting

There were 1867 patients who underwent CABG from July 2020 to December 2022 in Nanjing First Hospital. Of which 1309 patients got isolate CABG for first time (Detailed excluding process is shown in Fig. 1.). Totally 1247 patients used saphenous vein including 849 with OVH and 398 with EVH. In the original cohort, EVH group had a higher proportion of female, hypertension, hyperlipidemia, peripheral arterial disease, history of PCI, angina and acute coronary syndrome(all *P* < 0.05), meanwhile patients in EVH group had a higher body mass index, more triple-vessel disease and left main disease(all *P* < 0.05). After propensity score matching, 308 pairs successfully matched with baseline SMDs < 0.1 for all baseline characteristics (Table 1).

### In-hospital outcomes

The surgical information and postoperative in-hospital outcomes in the PSM groups are shown in Table 2. With the surgical information, patients in the EVH group had more numbers of vein graft (3 vs. 2, *P* < 0.001) and the OVH group had more intraoperative blood transfusion (19.5% vs. 35.3%, *P* < 0.001). There were no significant differences in operating time, cardiopulmonary bypass use and LIMA use between cohorts. EVH patients had more rates of acute kidney injury (8.5% vs. 4.2%, *P* = 0.045) and less prolonged mechanical ventilation (4.9% vs. 10.1%, *P* = 0.023). Death in hospital rates (2.3% vs. 1.3%, *P* = 0.543) and other in hospital postoperative outcomes (ICU stay time, low cardiac output, cardiac arrest, reoperation for bleeding and cerebral vascular accident) did not show the differences in the groups. The similar tendencies were observed between the cohorts before propensity matching (Supplementary Material, Table S1.)

**Table 2:** Surgical information and postoperative in hospital outcomes in propensity matched patients with open vein harvesting and endoscopic vein harvesting

	EVH group (*n* = 308)	OVH group (*n* = 308)	*P* value
Operating time, median(IQR), min	260(230,300)	265(225,300)	0.635
Cardiopulmonary bypass use, n(%)	264(85.7)	264(85.7)	0.840
CPB time, median(IQR), min	97(80,117)	96(82,115)	0.573
ACC time, median(IQR), min	62(50,77)	63(51,79)	0.756
Use of LIMA, n(%)	297(96.4)	297(96.4)	1.000
Numbers of vein graft, median(IQR), min	3(2,3)	2(3,4)	< 0.001
Intraoperative blood transfusion, n(%)	57(19.5)	108(35.3)	< 0.001
ICU time, median(IQR), day	1(1,2)	1(1,2)	0.119
Low cardiac output, n(%)	10(3.3)	12(3.9)	0.840
Cardiac arrest, n(%)	3(1.0)	3(1.0)	1.000
Reoperation for bleeding, n(%)	3(1.0)	5(1.6)	0.725
Readmitted to ICU, n(%)	6(1.9)	8(2.6)	0.787
Acute kidney injury, n(%)	26(8.5)	13(4.2)	0.045
Prolonged mechanical ventilation, n(%)	15(4.9)	31(10.1)	0.023
Cerebral vascular accident, n(%)	13(4.3)	16(5.2)	0.717
Death in hospital, n(%)	7(2.3)	4(1.3)	0.543

OVH: open vein harvesting. EVH: endoscopic vein harvesting. IQR: interquartile range. CPB: cardiopulmonary bypass. ACC: aortic cross clamping. LIMA: left internal mammary artery

### TTFM results in CABG

All patients received intraoperative TTFM during the surgery. There are totally 1189 saphenous vein grafts(SVGs) from 616 patients could be counted (664 in EVH group and 525 in OVH group, Table 3). Depending on differences of the target vessels, we categorized them as LAD system(mainly Diagonal), LCX system(including OM) and RCA system(including RCA, PL and PDA). Most SVGs showed well fluency with the averages of mean graft flow(MGF) over 30mL/min and PI < 3.0. The EVH grafts had higher MGF(39.6 mL/min vs. 35.9 mL/min, *P* = 0.022) than OVH grafts with nearly the same PI(2.3 vs. 2.4, *P* = 0.240). There were no significant differences in MGF between groups when categorize the SVGs with target vessels.

**Table 3:** Intraoperative transit time flow measurement between endoscopic and open vein harvesting patients in different target vessels

Target vessel	Variables	EVH group Mean(SD)	OVH group Mean(SD)	Difference*	95% CI	*P*-value
Diagonal/LAD (*n* = 173, 133)	MGF	35.2 (17.99)	34.4 (19.53)	0.56	-3.66, 4.78	0.796
	PI	2.0 (1.08)	2.6 (3.59)	-0.57	-1.14, -0.01	0.049
OM (*n* = 228, 183)	MGF	42.1 (26.32)	36.6 (26.87)	4.43	-0.65, 9.51	0.088
	PI	2.2 (1.22)	2.5 (1.69)	-0.27	-0.55, 0.01	0.062
RCA/PDA/PL (*n* = 263, 209)	MGF	40.4 (24.45)	36.4 (21.08)	3.41	-0.75, 7.57	0.109
	PI	2.5 (2.12)	2.3 (1.86)	0.26	-0.11, 0.62	0.170
Total (*n* = 664, 525)	MGF	39.6 (23.76)	35.9 (22.89)	3.10	0.45, 5.75	0.022
	PI	2.3 (1.62)	2.4 (2.37)	-0.14	-0.36, 0.09	0.240

*The differences were adjusted for the diameters of corresponding target vessel. MGF: mL/min

EVH: endoscopic vein harvesting. OVH: open vein harvesting. CI: confidence interval. MGF: mean graft flow. PI: pulsatility index. LAD: left anterior descending. OM: obtuse marginal. RCA: right coronary artery. PDA: posterior descending artery. PL: posterior branches of left ventricular

### Survival analysis

There were 19 patients who had in-hospital death with 10 in EVH group. The main reason of in-hospital death was ventricular arrhythmia(42.1%, 5/10 in EVH group, 4/9 in OVH group). The median (interquartile range) follow-up time was 1.5 (1.0–2.0) years in EVH cohort and 1.67 (1.0-2.25) years in OVH cohorts. Totally 28 patients were lost to follow-up (2.0%), and no significant difference was found between 2 cohorts (2.5% vs. 1.6%, *P* = 0.301).

**Fig. 2:**
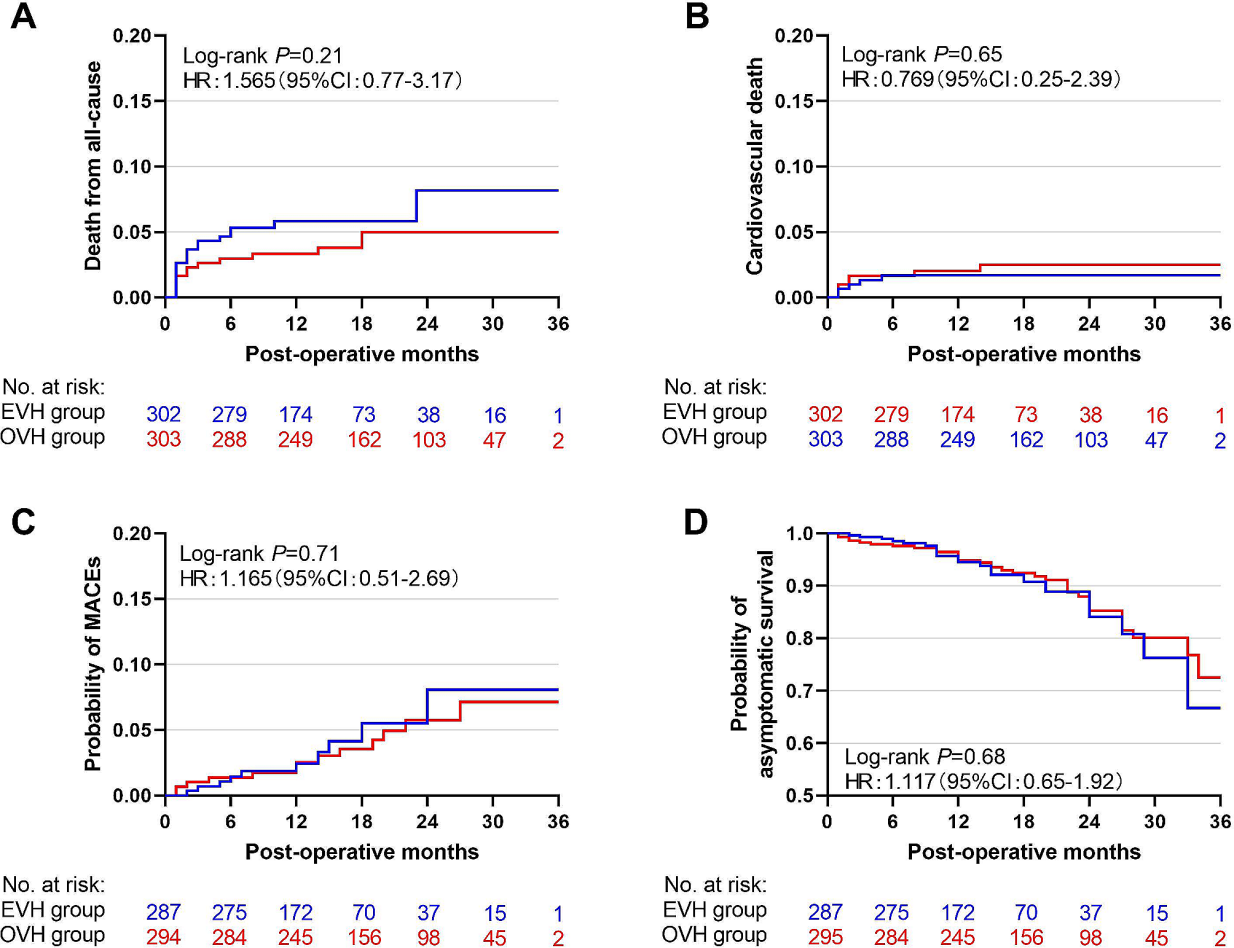
Survival curves at the mid-term follow-up. Cumulative incidence curves illustrating the mid-outcomes of the rates of all-cause death (**A**), cardiovascular death (**C**) and MACEs (**B**) in the propensity matched cohort of EVH (blue) and OVH (red) group. Kaplan-Meier curves showing the mid-outcomes of overall asymptomatic survival (**D**) between two groups. No significant differences were observed between EVH and OVH groups in mid-outcome rate of all-cause death (log-rank *P* = 0.21), cardiovascular death (log-rank *P* = 0.65). After the competing risk model, there were also no difference in MACEs (log-rank *P* = 0.71) and asymptomatic survival (log-rank *P* = 0.68) in mid-term follow-up showed no difference HR: hazard ratio. CI: confidence interval. EVH: endoscopic vein harvesting. OVH: open vein harvesting. MACEs: major adverse of cardiovascular events

The all-cause death rate had no significant difference in different vein harvesting during the 3 years follow-up. The rate of death from all cause was 8.5% in EVH cohort vs. 5.0% in OVH cohort (HR 1.565, 95%CI: 0.77–3.17, Log-rank *P* = 0.21) (Fig. 2A). After adjusting for death from non-cardiovascular causes as a competing risk, death from cardiovascular causes was 1.7% in EVH group vs. 2.5% in OVH group(HR 0.769, 95%CI: 0.25–2.39, Log-rank *P* = 0.65) (Fig. 2B).

As the secondary outcomes, no significant differences were observed in the incidence of MACEs(8.1% vs. 7.1%, HR 1.165, 95CI: 0.51–2.69, Log-rank *P* = 0.71)(Fig. 2C) and asymptomatic survival(66.7% vs. 72.5%, HR 1.117, 95%CI: 0.65–1.92, Log-rank *P* = 0.68)(Fig. 2D) after adjusting death as a competing risk. The same results were observed in the original cohorts (Figure S1).

## Discussion

In this retrospective cohort study, we found that comparing with open vein harvesting, the grafts conducted by endoscopic vein harvesting showed the comparable prognosis in isolated CABG patients in 3 years follow-up with the similar morality, rates of MACEs and asymptomatic survival.

EVH method have evolved rapidly and been widely used in the last two decades because of its smaller incisions. About 50–70% patients with CABG use EVH to conducts the vein grafts in western countries [12, 13]. In this study, EVH was performed in 32.0% patients which had a smaller percentage comparing with some previous studies. EVH is associated with a learning curve and the saphenous vein conducted by inexperienced surgeons could cause the early graft failure [14]. In this research, the EVH procedure was conducted by an experienced surgeon to decrease the bias caused by the surgery.

Two subgroup analysis of the clinical trials(PREVENT IV Trial and ROOBY Trial) suggested that EVH was associated with higher rates of vein graft failure and repeat revascularization [15, 16]. However, these two RCTs were conducted 10 years ago and were not designed to compare the outcomes of EVH and OVH technique. REGROUP Trial [7] was the first randomized trial to describe the outcomes between EVH and OVH in western countries. In our study, we compare the early and mid-term prognosis of EVH grafts which showed that there were no significant differences in the rates of all-cause death and MACEs in 3 years after the CABG surgery comparing with OVH groups. This is consistent with the results of several recent retrospective studies [17–19] and REGROUP Trial.

Currently, TTFM was more confirmed the association with graft patency and postoperative clinical outcomes [9]. Early graft failure occurred predominantly in SVGs with lower MGF and higher PI [20, 21]. In this study, most vein grafts measured by TTFM during the surgery. The MGF of all vein graft counted were over 31 ml/min, which was considered as a risk factor of early graft failure [22]. Meanwhile, we found that EVH grafts showed a higher MGF than OVH grafts and the PI between cohorts had no significant differences which suggested that appropriately stretching saphenous vein in EVH technique may not have the immediate effect on MGF in the surgery. This could also explain that similar prognosis in the early and mid-term follow-up.

There are still several limitations of this research. Firstly, this study was a single-center retrospective cohort study, some potential biases could not be fully avoided and a multicenter randomized controlled study could be more convincing. Secondly, EVH procedures were conducted by the same experience surgeon, however, the OVH were performed by different surgeons in our center, which could affect the efficiency of treatment in OVH group.

## Conclusion

In this cohort study from East China, EVH shows a similar in-hospital outcomes comparing with OVH. Also, in the 3 years follow-up, EVH is not associated with worse clinic outcomes(all-cause death, MACEs) to OVH. Longer follow-up need to be conducted further to evaluate the long-term prognosis of EVH additionally.

### Electronic supplementary material

Below is the link to the electronic supplementary material.

Supplementary Material 1

## Data Availability

No datasets were generated or analysed during the current study.

## Abbreviations

CABG: Coronary artery bypass graft
EVH: Endoscopic vein harvesting
LIMA: Left internal mammary artery
MACEs: Major adverse cardiovascular events
OVH: Open vein harvesting
TTFM: Transit time flow measurement
