# Supplementary material for: Mid-term outcomes of endoscopic vein harvesting in coronary artery bypass grafting: a retrospective cohort study
Source: J Cardiothorac Surg. 2024 Jun 27;19:389. doi: 10.1186/s13019-024-02930-5 (PMC11210013; doi:10.1186/s13019-024-02930-5)
Supplement: Supplementary file 1 — Supplementary Material 1 [file 13019_2024_2930_MOESM1_ESM.docx]

**Table S1**. Surgical information and postoperative in hospital outcomes with open vein harvesting and endoscope vein harvesting before propensity matching.

|  | EVH group  (n=398) | OVH group  (n=849) | *P* value |
| --- | --- | --- | --- |
| Operating time, median(IQR), min, n=1217 | 260(230,300) | 260(225,300) | 0.451 |
| Cardiopulmonary bypass use, n(%) | 339(85.2) | 724(85.3) | 1.000 |
| CPB time, median(IQR), min, n=1051 | 97(79,117.5) | 96(79,116) | 0.387 |
| ACC time, median(IQR), min, n=1049 | 62(49,77) | 63(50,78) | 0.856 |
| Use of LIMA, n(%) | 386(97.0) | 817(96.2) | 0.611 |
| Numbers of vein graft, median(IQR), min | 3(2.3) | 2(2.3) | <0.001 |
| Intraoperative blood transfusion, n(%), n=1216 | 71(18.9) | 293(34.9) | <0.001 |
| ICU time, median(IQR), day | 1(1,2) | 1(1,2) | 0.210 |
| Low cardiac output, n(%), n=1240 | 14(3.5) | 33(3.9) | 0.880 |
| Cardiac arrest, n(%), n=1240 | 6(1.5) | 8(0.9) | 0.394 |
| Reoperation for bleeding, n(%), n=1237 | 5(1.3) | 18(2.1) | 0.414 |
| Readmitted to ICU, n(%) | 9(2.3) | 20(2.4) | 1.000 |
| Acute kidney injury, n(%), n=1237 | 34(8.6) | 44(5.2) | 0.031 |
| Prolonged mechanical ventilation, n(%), n=1239 | 23(5.8) | 70(8.3) | 0.155 |
| Cerebral vascular accident, n(%), n=1240 | 18(4.6) | 38 (4.5) | 1.000 |
| Death in hospital, n(%) | 10(2.5) | 9(1.1) | 0.088 |


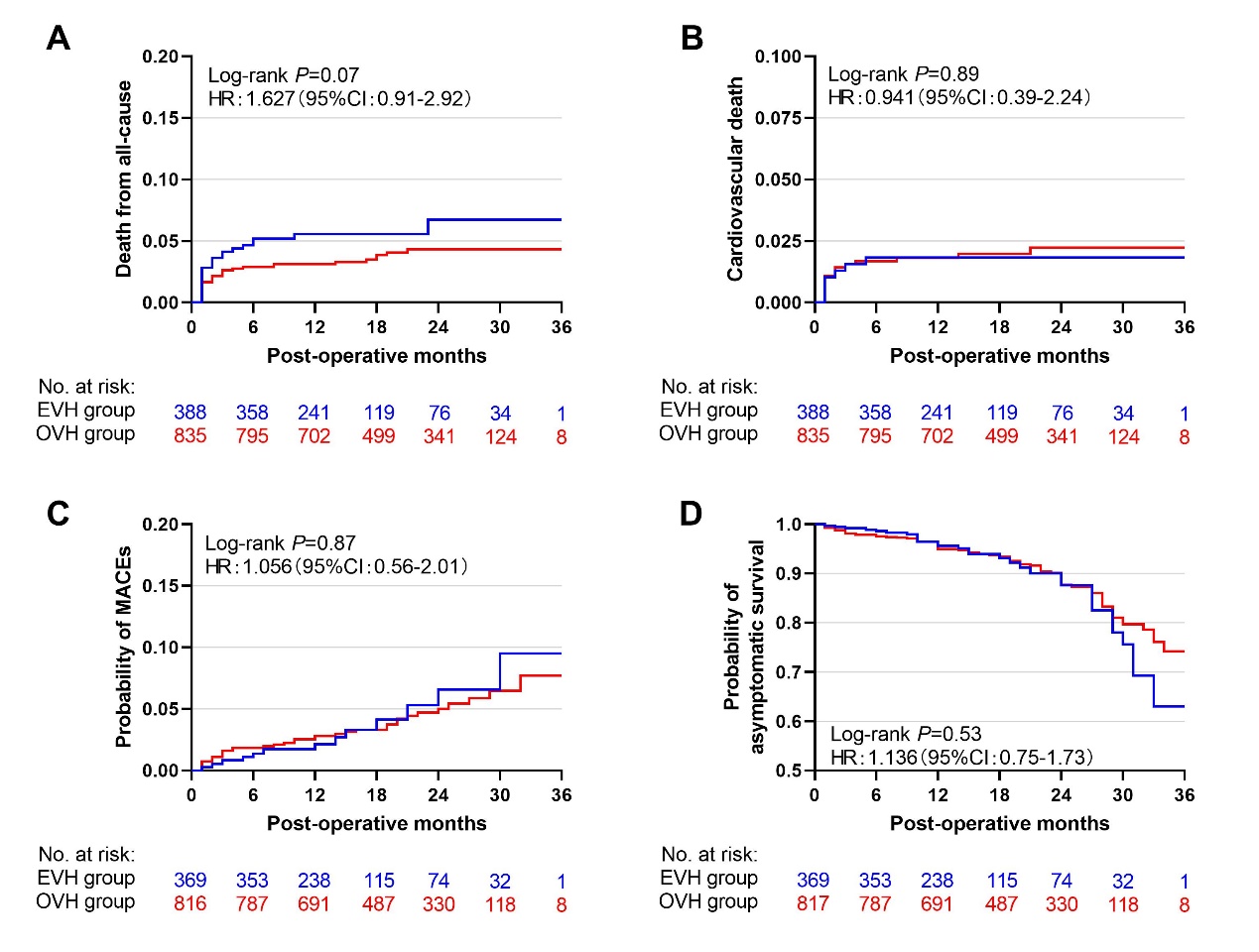


**Figure S1. Survival curves at the mid-term follow-up before propensity matching.**

Cumulative incidence curves illustrating the mid-outcomes of the rates of all-cause death (A) , cardiovascular death (B) and MACEs (C) in between EVH(blue) and OVH(red) group before propensity matching. Kaplan-Meier curves showing the mid-outcomes of overall asymptomatic survival(D) between two groups. There were no significant differences in all-cause death, cardiovascular death, MACEs incidence and asymptomatic survival between groups(all *P*>0.05).

HR: hazard ratio. CI: confidence interval. EVH: endoscopic vein harvesting. OVH: open vein harvesting. MACEs: major adverse of cardiovascular events.
